# Comparison of Antimicrobial Resistance in Escherichia coli Strains Isolated From Swine, Poultry, and Farm Workers in the Respective Livestock Farming Units in Greece

**DOI:** 10.7759/cureus.51073

**Published:** 2023-12-25

**Authors:** Magdalini K Christodoulou

**Affiliations:** 1 School of Health Sciences, University of Thessaly, Larissa, GRC

**Keywords:** greece, minimum inhibitory concentration (mic), antibiotic resistance, e. coli, farmers, poultry, swine

## Abstract

Human health is at risk because commensal intestinal bacteria in livestock have been developing increased antibiotic resistance, mainly due to antibiotics’ extensive use in recent years. In this study, we compared the antibiotic resistance of *E. coli* strains isolated from fecal samples obtained from swine, poultry, and farm workers in the corresponding livestock farming units. The study aimed to investigate the correlation between the antibiotic resistance patterns of *E. coli* in livestock and in humans closely interacting with them. Antibiotic resistance is determined using the microdilution method, which measures the minimum inhibitory concentration (MIC) for seven commonly used antimicrobial agents. Most *E. coli* isolates displayed significant resistance to tetracycline and ampicillin. Resistance to sulfamethoxazole was observed, too, in swine and swine farmers. In contrast, high resistance rates to nalidixic acid were observed in *E. coli* strains isolated from poultry and poultry farmers, with percentages of 66.7% and 50%, respectively. Furthermore, 31.57% of the isolated strains from swine and swine farmers were resistant to at least one antibiotic. In comparison, 44.44% of the poultry strains and 33.33% of the poultry farmers' strains were resistant to at least two antibiotics. Additionally, a high prevalence of multidrug resistance was observed among the *E. coli* strains isolated from all four categories. The study’s results provide evidence that the use of antibiotics and the increased resistance of intestinal bacteria affect the resistance of intestinal bacteria in people working on farms. These findings highlight the potential role of antibiotic use in animals as a contributing factor to the development of antibiotic resistance in both animals and humans. Additionally, it suggests that individuals working on farms may be at an increased risk of acquiring antibiotic-resistant strains of *E. coli* due to their proximity to and interaction with animals.

## Introduction

Antimicrobial resistance (AMR) is a global concern that jeopardizes the effective treatment of bacterial infections in both humans and animals [1]. In animal agriculture, the misuse and overuse of antibiotics have contributed to the emergence and spread of antimicrobial-resistant bacteria [2]. Antibiotic use exerts selective pressure, promoting the survival and spread of resistant bacteria in animals and humans, raising significant concerns for prevention and treatment [3]. Moreover, the unregulated administration of antibiotics to animals raised for food production is concerning not only in terms of veterinary clinical implications but also due to the potential for zoonotic transmission, posing risks to human health [4]. Many studies revealed high rates of antibiotic resistance among *E. coli* strains isolated from pigs and poultry, with resistance observed against tetracyclines, ampicillin, sulfamethoxazole, and nalidixic acid [5]. Similar findings have been reported in studies conducted in Europe and the USA, highlighting the global nature of this problem [6]. Osterberg et al. emphasize the uncontrolled use of antibiotics in pig production, where antibiotics are extensively utilized for growth promotion, prophylactic treatments, and therapeutic purposes [7]. This study aims to examine the occurrence of antibiotic resistance in *E. coli* isolates from the feces of healthy workers on pig and poultry farms and to contrast this information with isolates collected from pigs and poultry on the same farms.

## Materials and methods

The study was conducted during the spring months of 2020. We investigated antibiotic resistance in Greece by sampling fecal specimens from 18 swine farms and 19 poultry farms in the regions of Thessaly, Central Greece, and Macedonia. Samples were collected from prefectures in Greece, including Pieria (three samples), Thessaloniki (11 samples), Fthiotida (17 samples), Karditsa (three samples), Larissa (one sample), and Fokida (two samples). Based on their geographic location within the regions of Macedonia, Thessaly, and Central Greece, pig and poultry farms were chosen for sampling.

Farms were chosen to ensure a representative sample of the different areas in each region. The inclusion criteria for the swine farms included a minimum herd size of 50 animals and a history of antibiotic usage. For poultry farms, inclusion criteria consisted of a minimum flock size of 500 birds and a history of antibiotic administration. This sampling approach aimed to capture a range of farming practices and potential variations in antibiotic resistance profiles.

Our objective was to create pairs of samples from animals and workers, and our goal was to compare the antibiotic resistance profiles of the isolated *E. coli* strains in different geographical areas of the country.

The study was conducted in accordance with relevant national and international guidelines. According to national legislation, ethical approval is not required when animals are not subjected to any manipulation and when the owner of the animal has given their consent.

The study adhered to strict ethical guidelines to protect the participants and ensure the scientific integrity of the research. Specifically, the study obtained approval from the ethics committee of the University of Thessaly before its commencement.

The farmers who participated in the study signed a consent form, thereby expressing their full consent to participate in the research. Furthermore, the employees who took part were thoroughly informed about the study and their participation, which was communicated through telephone or email.

Collection of fecal samples

Collection of Samples From Swine and Poultry

A fresh sample of feces was collected randomly from each animal for analysis. Fattening pigs and poultry were selected for sampling because they are usually given antibiotics added to their food or water for treatment, growth promotion, or prevention. This frequent exposure to antibiotics increases the likelihood of the development of antibiotic-resistant bacteria, which can easily transfer among them due to close contact. The aim of this study is to investigate the prevalence of antibiotic-resistant bacteria in the fecal samples of fattening pigs and poultry.

Collection of Fecal Samples From Swine and Poultry Farmers

Fecal samples were collected exclusively from apparently healthy workers who had not received antibiotic treatment in the last three months. They completed a consent agreement and a relevant questionnaire with epidemiological data (gender, age, any medication) both for themselves and for the animal from which the sample was taken. Each sample was coded so the confidentiality of the worker’s data could be respected and assured.

Both animal and human samples were carefully placed in sterile containers and promptly transported, within a span of fewer than 24 hours, under refrigeration in insulated containers to the Hygiene Laboratory and Epidemiology Department of the University of Thessaly.

Isolation and identification of *E. coli*

The following procedures were carried out to isolate and identify *E. coli* in laboratory tests. Upon arrival at the laboratory, the stool samples were analyzed microbiologically within 24 hours.

Stool Suspension Creation

A sterile 15-ml tube (Falcon 15-ml) was used to add 10 ml of sterilized water and 2 μl of stool sample under aseptic conditions. The suspension was created through a mechanical shaker in a sterile container in order to remove any solid material.

Inoculation on Selective Nutrient Mediums

Inoculation on selective nutrient mediums: 10μl of the suspension was aseptically transferred onto the surface of MacConkey Agar (MAC) medium from "Biolife." The MAC medium contains lactose, which inhibits the growth of many Gram-positive microorganisms without inhibiting the growth of *enterobacteria* and controls lactose fermentation. The medium was prepared by dissolving 55g of dehydrated medium in 1000 ml of deionized water. The medium was then coated using a sterile loop under aseptic conditions. Incubation was followed at 37°C for 24 hours under aerobic conditions. Afterward, the solution was mixed until boiling and sterilized at 121°C for 15 minutes. After sterilization and cooling to 45-50°C, the liquid medium was distributed into sterilized Petri dishes under sterile conditions and solidified. The prepared Petri dishes with SMAC were stored under refrigeration at 4°C until further use.

Subculturing of Colonies on Nutrient Agar (NA)

Suspicious colonies were subcultured on NA. Incubation was followed at 37°C for 24 hours. The NA was divided into three portions (a, b, and c) so that each portion could be used to cultivate a colony to further isolate *E. coli*.

Biochemical Analyses

An oxidase test was performed for each isolated colony. 2-3 drops of the oxidase reagent from «Biomerieux» were placed on filter paper. A small amount of the suspicious colony was streaked onto the filter paper using an inoculation loop. The appearance of a blue color indicated a positive result (presence of the cytochrome oxidase enzyme). Oxidase-positive bacteria were discarded. The oxidase-negative ones were placed in tryptone water and incubated at 44.5°C for 24 hours. Tryptone (tryptophan) broth from Bio Merieux was prepared by dissolving 15g of dehydrated medium in 1000 ml of deionized water. The solution was divided into test tubes and sterilized at 121°C for 15 minutes. After incubation, the indole test was performed, which relies on the ability of *E. coli* to oxidize tryptophan in the presence of oxygen to form indole. Drops of Kovac's reagent were added to the tube containing tryptone water. A red ring formation on the liquid's surface indicated the presence of indole, while the absence of a ring resulted in a yellow color. Indole-positive strains were further tested for their basic biochemical properties using the API 20E test (Bio Merieux, France), a standardized system consisting of 20 microtubes containing dehydrated substrates for enzymatic activity or sugar fermentation. The microtubes were inoculated with a dense suspension of microorganisms derived from a pure culture. During incubation at 34-38°C for 18-24 hours, metabolic changes caused visible color reactions, either automatically or upon the addition of specific reagents. A comparison was made between the observed color changes in each API 20E strip and those recorded in the database, and the identification of the tested microorganism species was determined accordingly. The four IMVC tests (indole, methyl red, Voges-Proskauer, and citrate) are internationally recognized as the indole production, methyl red, Voges-Proskauer, and citrate utilization tests. For *E. coli*, the results of these tests are IMVC: + + - -.

Preservation of Identified E. coli Strains

The identified *E. coli* strains were preserved in deep freezing at -80°C in glycerol for further antimicrobial resistance testing.

Antimicrobial susceptibility test

The antibiotic resistance of *E. coli* strains was evaluated for seven antibiotics: ampicillin (AM), chloramphenicol (CHL), gentamicin (GE), neomycin (NE), sulfamethoxazole (SUL), tetracycline (TE), and nalidixic acid (NAL). The susceptibility of the bacteria was determined by assessing the minimum inhibition concentration (MIC) for each antibiotic using the successive microdilution technique on microtiter plates. The testing was carried out according to the guidelines of the Clinical and Laboratory Standards Institute (CLSI) [8]. The following steps were taken:

Preparation of Microbial Suspension

A concentration of 1-2 x 10^8 CFU/ml for *E. coli* strain ATCC 25922 was obtained using a turbidity standard of 0.5 on the McFarland scale. Approximately seven colonies from a recent subculture were selected using a cotton swab and mixed with 10 ml of saline solution. The turbidity of the suspension was adjusted using the turbidity standard. An inoculum suspension was then prepared by diluting the initial suspension 1:10 (*E. coli* concentration of 15 x 10^6 CFU/ml) in Mueller-Hinton broth.

Technique of Antibiotic Dilution in Broth

The microdilution technique was used, which involves using small volumes (0.05-0.2 µl) in special microtiter plates. This technique allows simultaneous testing of multiple different concentrations of selected antibiotics. The procedure for creating the antibiotic dilutions was as follows:

Preparation of Microplates

Twofold dilutions of the antibiotics in Mueller-Hinton broth were prepared. Ten dilutions were defined on either side of the concentration values recommended by the CLSI for determining *E. coli* sensitivity to each antibiotic. In each microplate row, dilutions of the same antibiotic were added, starting from the lowest to the highest concentration. Then, 50 µl of the antibiotic solution was added to each well, ensuring that the antibiotic concentration in each well was twice the desired concentration. From the final microbial suspension prepared, 50 µl was added to each well containing the antibiotic. The microplates were covered to prevent evaporation and incubated under aerobic conditions at 37°C for 18-24 hours.

Results

After incubation, microplates were examined on a viewing device with an enlarging mirror and indirect light. Bacterial growth was detected as a pellet at the bottom of the well. The first well without growth was considered the minimum inhibitory concentration (MIC) of the tested antibiotic and was recorded. In some cases, two consecutive wells with growth in a two-fold dilution series, followed by a dilution without growth, were observed. In such cases, the method was repeated. Conversely, two cases with a single well without growth were disregarded. For each strain, the concentration of the microbial suspension with the lowest antibiotic dilution, in which no growth was observed, determined the MIC for that antibiotic. *E. coli* ATCC 25922 was used as the reference strain.

Statistical analysis

The data collected underwent statistical analysis using the chi-squared test. The results of the antimicrobial susceptibility tests were presented as percentages or frequencies of animal or human isolates. A comparison was made between the percentages or frequencies of resistance in animal and human *E. coli* isolates. In all cases, a p-value of less than 0.005 was considered statistically significant.

## Results

Table 1 shows the results of a study on *E. coli* bacteria in pigs and workers. Of the 19 pig isolates tested, six (31.57%) were resistant to at least one antibiotic, with the numbers rising to three (15.78%) for two antibiotics, four (21%) for three antibiotics, and three (15.78%) for four or more antibiotics. Of the 18 poultry isolates tested, two (11.11%) were resistant to at least one antibiotic, with the numbers rising to eight (44.44%), seven (38.88%), and one (5.26%) for two, three, and four or more antibiotics, respectively. Both pig and poultry workers were also tested. Of the 19 pig worker isolates tested, six (31.57%) were resistant to at least one antibiotic, with the numbers rising to five (26.3%) for two antibiotics and four (21%) for four or more antibiotics. Of the 18 poultry worker isolates tested, four (22.22%) were resistant to at least one antibiotic, with the numbers rising to six (33.33%), two (11.11%), and five (27.21%) for two, three, and four or more antibiotics, respectively. The results from the pig and pig worker isolates were not significantly different from one another in terms of resistance percentages and multi-drug resistance percentages. Resistance to tetracycline, amoxicillin, and ampicillin was detected at the highest frequency in *E. coli* isolates from pigs and pig workers. The poultry isolates showed high resistance to tetracycline, nalidixic acid, and neomycin, with lower levels of resistance to ampicillin, sulfamethoxazole, and chloramphenicol. Notably, no resistance to gentamicin was found among these strains. Conversely, among the strains isolated from poultry farmers, the highest resistance was observed for tetracycline and ampicillin. A statistically significant difference was observed only in ampicillin resistance between avian and poultry farmer strains, with a p-value of 0.01, indicating statistical significance. The calculated R.R. of 0.33 suggests that strains isolated from avian species have a 66% lower likelihood of being sensitive to ampicillin than those isolated from poultry farmers. The findings suggest that antibiotic resistance (Table 2) is present in both pig and poultry populations and their associated workers. Comprehensive surveillance and control measures are necessary to combat the spread of multidrug-resistant strains in livestock and workers.

**Table 1:** Antibiotic resistance of E. coli isolated from pigs and pig farm workers.

Antimicrobial agent	Break point mg/L	Pigs n = 19		Workers n = 19	
		N	% resistance	N	% resistance
Ampicillin	>8	6	31.6	10	52.6
Sulfamethoxazole	>256	7	36.8	3	15.8
Nalidixic Acid	>16	4	21.1	3	15.8
Neomycin	>8	2	10.5	1	5.3
Tetracycline	>8	16	84.2	14	73.7
Gentamycin	>8	0	0.0	0	0
Chloramphenicol	>16	1	5.3	2	10.5

**Table 2:** Antibiotic resistance of E. coli isolated from poultries and poultry farm workers.

Antimicrobial agent	Break point mg/L	Poultries n = 18		Workers n = 18	
		N	% resistance	N	% resistance
Ampicillin	>8	4	22.2	12	66.7
Sulfamethoxazole	>256	3	16.7	3	16.7
Nalidixic Acid	>16	12	66.7	9	50
Neomycin	>8	6	33.3	3	16.7
Tetracycline	>8	15	83.3	12	66.7
Gentamycin	>8	0	0.0	3	16.7
Chloramphenicol	>16	1	5.6	4	22.2

## Discussion

The poultry and swine industry is essential for global food security as it provides a significant source of animal protein. However, the overuse of antibiotics has led to antimicrobial resistance in intestinal bacteria found in food animals [9,10]. This has become a significant threat to the livestock environment across the world. The antibiotic resistance of *E. coli* isolates that we found in pigs was higher than that of the same bacteria found in pig farm workers. Similar patterns have been documented in other studies as well [11]. Most of the *E. coli* strains that are isolated from swine and pig farm workers show high resistance to ampicillin, tetracycline, and sulfamethoxazole in both humans and livestock. These results are consistent with those of similar studies conducted in other countries [12,13].

The overuse of antibiotics in animal treatment and productivity enhancement has led to a significant increase in antibiotic resistance [14]. This is particularly relevant for tetracyclines, which have been extensively used in veterinary therapy, prevention, and growth promotion in pigs, among others [15]. Tetracyclines have been widely used for bacterial infections, but their effectiveness has been reduced due to the evolution of resistance mechanisms [16]. These mechanisms include the presence of TET genes that encode efflux pumps and ribosome-protecting proteins, as well as the enzymatic degradation of the antibiotics. Efflux pumps are a common resistance mechanism in both gram-negative and gram-positive bacteria, including *E. coli* strains from pigs that show resistance to tetracyclines [17].

The majority of *E. coli* strains from pigs resistant to tetracyclines (TET-R) were also resistant to two or more antibiotics. Specifically, 24% showed resistance to ampicillin-sulfonamides and tetracyclines (AMP-SUL-TET) [18]. Additionally, population density is a crucial factor in the dissemination of resistant bacteria and resistance between individuals. This contributes to the further increase of antibiotic resistance in a population [19].

A considerable percentage of *E. coli* strains isolated from swine have been observed to possess resistance against numerous antibiotics, with tetracycline resistance being the most commonly observed. The increased resistance of strains of *E. coli* to sulfamethoxazole (sulfonamides) can be attributed to the historical use of this antibacterial agent in pigs to control respiratory diseases and as a growth promoter through their food, which has created selective pressure and favored the emergence and spread of resistant bacterial clones [20]. This resistance has been observed in various countries, including Switzerland, the United States, and Canada [18,20]. It is possible that the use of antimicrobial agents in swine farms and intensive farm management practices contribute to the transmission of antibiotic-resistant bacteria in swine hosts and the farm environment. It is likely that resistant bacteria can be transferred from food animals to humans through occupational exposure or waste runoff from animal production facilities. This is because the bacteria can colonize humans upon contact. The results of several studies suggest that commensal bacteria that are resistant can be transmitted from animals to humans and vice versa [21].

The study examined 18 pairs of *E. coli* strains from poultry and poultry farmers. The results showed that tetracyclines, quinolones, and neomycin exhibited the highest rates of resistance, with tetracyclines, ampicillin, and nalidixic acid showing the highest rates of resistance in poultry strains. These findings are consistent with previous studies that have established a link between antibiotic use and bacterial resistance in *E. coli* [22].The prevalence of antimicrobial resistance among fecal *E. coli* isolates may be attributed to the transmission of resistant bacteria or resistance plasmids from poultry to poultry workers [23]. The selection of antimicrobial resistance in bacteria is primarily attributable to the misuse of antibiotics, overcrowding, and poor sanitation practices in poultry farms [24]. These attributes are commonly associated with intensive poultry farming and can explain the high prevalence of resistance to fecal *E. coli* among poultry workers in this study as well as in other similar studies [25].

The study reports the presence of multiple resistances to penicillins and sulfonamides. The resistance to sulfamethoxazole suggests the existence of class I integrons, which are significant contributors to the development of resistance against multiple antimicrobial agents [26]. Poultry farmers show a notable prevalence of neomycin resistance, which could be due to the use of neomycin-containing ointments for occupational cuts and skin lesions [27]. It is noteworthy that the increased resistance of *E. coli* to nalidixic acid can be attributed to the use of quinolones in poultry for therapeutic purposes as well as the treatment of infections in poultry farmers. The high resistance rate among strains isolated from poultry farmers can also be due to the fact that more than 50% of the sample was composed of individuals over 55 years of age. This is because the prior exposure of a patient to a fluoroquinolone is the most significant risk factor for developing a fluoroquinolone-resistant *E. coli* infection [28].

The regulations in Greece regarding the use of antimicrobials in farm animals are consistent with those of the European Union (EU), which prohibits the use of all antimicrobials as growth promoters to prevent the emergence of antimicrobial resistance. Despite the need for a clear definition of "therapeutic" versus "non-therapeutic" use of antibiotics, there are still instances where antibiotics are inappropriately administered under the guise of "therapeutic" purposes [29].

Antibiotic resistance in food animals not only poses a threat to animal health, but it also has significant implications for human health. The presence of antibiotic-resistant bacteria in food animals can be transmitted to humans through food consumption or occupational exposure, leading to difficult-to-treat infections in humans, increased healthcare costs, and higher mortality rates. Furthermore, the emergence of antibiotic resistance in food animals can compromise the effectiveness of antibiotics in treating both animal and human diseases, thereby jeopardizing global food security and public health.

Some potential solutions to address antibiotic resistance in food animals include implementing stricter regulations on the use of antibiotics in animal farming, promoting the responsible use of antibiotics in veterinary medicine, and investing in alternative approaches to disease prevention and treatment, such as vaccines and probiotics. Additionally, improving hygiene practices in animal farming and promoting better biosecurity measures can help reduce the spread of antibiotic-resistant bacteria among food animals. By implementing these measures, it is possible to mitigate the emergence and spread of antibiotic resistance in food animals and protect both animal and human health.

Limitations

During the execution of the study, several limitations were encountered. The primary challenge was obtaining cooperation from workers in livestock units due to their lack of knowledge or unwillingness to provide information on animal diet and medication administration. Moreover, some worker samples were collected on different days, which could impact the reliability of the results and lead to their exclusion from analysis. Lastly, the completion of questionnaires was limited due to inadequate cooperation. Despite these limitations, we hope that our study contributes to the field. We urge greater collaboration between workers and researchers to improve livestock management practices and ensure animal welfare.

## Conclusions

The emergence of antibiotic resistance in *E. coli* strains affecting humans is significantly linked to the use of antibiotics in animals. Recent research findings suggest that the use of antibiotics in animals has a direct impact on the development of antibiotic resistance in *E. coli* strains found in humans. This resistance pattern is similar in both animals and humans, which increases the risk of transmitting antimicrobial-resistant bacteria to humans. To address this issue, a cautious approach to the use of antimicrobial agents in food animals is recommended. It is important to minimize their usage whenever possible to reduce the selection and dissemination of resistant bacterial strains. Proper farm management practices and hygiene measures are also crucial to minimize the potential transmission of antimicrobial-resistant bacteria from animals to humans. To effectively combat microbial resistance to antimicrobial drugs, a comprehensive approach is required. This includes surveillance and recording of antibiotic consumption, adherence to sound veterinary practices, education of veterinarians and animal breeders, vaccination programs, implementation of disease control and eradication measures, as well as maintaining high standards of hygiene, biosecurity, and responsible management of livestock. Furthermore, alternative treatment approaches beyond the use of antibiotics should also be explored.
